# Introduction to Advances in nanophotonics, plasmonics, and nano-optics

**DOI:** 10.1039/d5na90067k

**Published:** 2025-10-08

**Authors:** Viktoriia E. Babicheva, Yu-Jung Lu, Alexander Shalin, Dattatray Late

**Affiliations:** a Department of Electrical and Computer Engineering, University of New Mexico, MSC01 11001, University of New Mexico Albuquerque New Mexico 87131 USA vbb@unm.edu; b Research Center for Applied Sciences, Academia Sinica Taipei City 115201 Taiwan; c Suzhou City University 1188 Wuzhong Blvd, Wuzhong District Suzhou Jiangsu 215104 China; d Department of Physics, Federal University of Lavras Campus Universitário, PO Box 3037 Lavras Minas Gerais 37200-000 Brazil datta099@gmail.com

## Abstract

Viktoriia E. Babicheva, Yu-Jung Lu, Alexander Shalin, and Dattatray Late introduce the *Nanoscale Advances* themed issue on Advances in nanophotonics, plasmonics, and nano-optics.
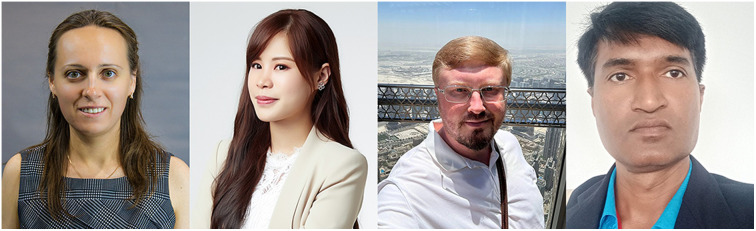

Nanophotonics, plasmonics, and nano-optics have entered a transformative stage, where fundamental theories are rapidly translating into experimental advances and device-level realizations, enabling unprecedented control over light at the nanoscale. Recent progress encompasses the design of optical nanoantennas, novel nanostructures, and self-organized composites that significantly enhance light–matter interactions, leading to breakthroughs in near-field optics, nonlinear responses, and quantum photonics. These developments have expanded the possibilities of spectroscopy, microscopy, and advanced imaging, offering new means of probing material properties with high precision. Research has also uncovered anomalies in light scattering and transport in nanomaterials, deepening our understanding of optical response in complex systems. With advancements in nanofabrication techniques, nano-optical trapping, and ultrafast optically induced magnetism, nanophotonics is pushing the boundaries of sensing, information processing, and functional material design, establishing itself as a cornerstone of modern optical science.

Polarisation imaging extends conventional imaging by revealing surface and material properties that remain hidden to standard intensity or colour techniques, with full-Stokes measurements enabling advanced data processing for remote sensing. An ultra-thin topology-optimised diffractive metasurface that encodes polarisation states into five diffraction orders within a telescope pupil plane is introduced, allowing for single-shot full-Stokes acquisition on a single detector [https://doi.org/10.1039/D5NA00298B]. The design provides stitched wide-field coverage, simultaneous error monitoring, and a compact, lightweight solution that overcomes the size and weight constraints of small-satellite platforms.

Rare-earth-doped phosphors are widely investigated for their structural, optical, and catalytic properties, with a particular interest in their potential applications in solid-state lighting and photocatalysis. A Dy^3+^-doped sodium zinc molybdate phosphor has been synthesised *via* a green microwave-assisted route, confirmed by P-XRD to crystallise in a monoclinic *C*2/*m* structure [https://doi.org/10.1039/D4NA00960F]. Photoluminescence studies revealed a dominant excitation at 348 nm and a sharp emission at 590 nm from the ^4^F_9/2_ → ^6^H_13/2_ transition, with optimal intensity at 6 mol% Dy^3+^. The phosphor exhibited orangish chromaticity and enhanced UV-driven photocatalytic activity, demonstrating multifunctional performance.

Conventional microwave-based techniques face fundamental limitations in characterizing nano-antennas at frequencies above a few terahertz, restricting progress in the study and design of plasmonic antennas. A new methodology has been introduced that employs electron energy loss spectroscopy (EELS) to determine input impedance and scattering parameters from the mid-infrared to optical range. A theoretical framework has been developed to relate EEL probability to microwave scattering parameters, which has been validated through experiments and simulations on a single plasmonic dipole [https://doi.org/10.1039/D4NA00960F]. The approach enables the extraction of *S*-parameters and impedance across the 25–150 THz range, offering a non-contact alternative to VNA-like characterization at otherwise inaccessible frequencies.

Surface plasmon interference has long served as a basis for optical metrology and sensing. In [https://doi.org/10.1039/D4NA00862F], a novel interferometer has been presented that employs paired grating couplers to excite surface plasmon polaritons along a gold–air interface before their convergence at a nanoslit. The interference response has been controlled by tailoring nanoslit geometry to support multiple resonance modes. Experimental validation has confirmed that adjusting coupler and nanoslit parameters has enabled tunable transmission. Furthermore, grating-induced focusing and far-field interference patterns have been demonstrated.

Surface-enhanced Raman spectroscopy has been recognized as a powerful technique for ultrasensitive molecular analysis, eliminating the need for selective binding. In [https://doi.org/10.1039/d4na00742e], a comprehensive workflow for SERS substrate development has been established using two-photon polymerization. Individual nanopillars with high aspect ratios have been fabricated by direct laser writing, enabling substantial field enhancement. The substrates have exhibited Raman signal amplification on the order of 10^6^, rivaling the performance of commercial devices. Prototyping has been completed within minutes to hours, and reproducibility has been achieved through the uniform fabrication of nanostructure arrays.

Kagome metals have attracted attention as platforms for exploring correlated and topological charge dynamics. A systematic investigation of FeSn has been performed in [https://doi.org/10.1039/D4NA00737A], revealing that optical conductivity is governed by two Drude responses originating from Dirac and massive bands, with the Dirac channel dominating at low temperatures. Cooling has shifted the spectral weight to lower frequencies as Dirac carrier mobility has risen due to reduced electron–phonon scattering. Interband dielectric measurements have matched density functional calculations, and the charge response of FeSn has been fully characterized for prospective photonic and plasmonic applications.

Metalenses have emerged as flat optical components capable of replacing conventional curved lenses by offering precise light control in a compact form. An artificial intelligence framework has been developed in [https://doi.org/10.1039/D5NA00550G] to predict the transmission and phase behavior of nanorod-based metasurfaces. Transfer learning has been applied using datasets of gallium nitride and titanium dioxide nanopillars on silica, with accuracy confirmed through experimental validation. The method has achieved low mean squared errors across the visible range, and focusing tests with two flat lenses have demonstrated practical viability. Angle-dependent response has also been assessed, showing potential for broader optical device design.

Surface-enhanced Raman scattering is renowned for its sensitivity; however, its reliability has often been limited by signal fluctuations related to substrate quality, polarization, and molecular distribution. A plasmonic chip has been fabricated in [https://doi.org/10.1039/D4NA00387J] to overcome these constraints by combining a periodic metallic grating with gold nanocubes, forming a 3D metasurface. Polarization-independent ultrahigh enhancement has been achieved, while signal averaging and normalization have reduced variability. The platform has enabled multimodal sensing through SERS, SEF, and SPR. Tests with rhodamine 6G and picric acid have demonstrated strong enhancement factors and a detection limit down to 3 nM.

Artificial intelligence has become a key enabler in the development of advanced photonic materials, offering unprecedented possibilities for manipulating light. Photonic crystals, plasmonic structures, and metamaterials have been engineered to achieve tailored optical responses, with PhCs drawing particular attention due to their self-assembly capabilities and distinctive properties such as bandgaps, reflectance, and visible-range lasing [https://doi.org/10.1039/D5NA00449G]. Recent works have reported progress in cost-effective fabrication of 3D nano-PhCs, where AI has been utilized to optimize design and manufacturing. Enhanced lasing performance and miniaturized optoelectronic devices have been demonstrated, highlighting promising applications in energy harvesting and intelligent systems.

Two-dimensional semiconductors have been widely explored as building blocks for next-generation light-emitting devices due to their tunable optical properties. A dual-colored emitter has been realized by applying AC carrier injection through separate electrodes with a controlled phase delay, allowing balanced output from WSe_2_ and WS_2_ monolayers [https://doi.org/10.1039/D5NA00623F]. This method has provided stable, spectrally distinct emissions with dynamic controllability. A balanced operation between the two materials has been achieved, and the approach has expanded opportunities for compact, multicolor optoelectronics, with implications for integrated photonic circuits and high-resolution display technologies.

Surface-enhanced Raman scattering has remained a subject of active investigation since its discovery more than five decades ago, with several fundamental aspects still under exploration. Recent experiments have revealed distinctive axial variations of SERS profiles by scanning the laser focus relative to a planar nanostructured substrate. Signal strength has exhibited Lorentzian behavior along the *Z* axis, with maxima consistently occurring above the surface [https://doi.org/10.1039/D4NA00982G]. Ratios between spectral components have also varied with axial position. Finite-difference time-domain simulations have attributed these effects to plasmonic near-field interactions, underscoring the importance of focus precision in quantitative SERS measurements.

Flexible sensing platforms have garnered interest due to their lightweight, adaptable nature and ability to conform to complex surfaces, providing advantages for detection in diverse environments. A plasmonic metamaterial sensor has been fabricated by nanoimprinting silver-coated polyvinylidene fluoride film, producing nanoscale arrays that act as polarization channels to support resonant enhancement [https://doi.org/10.1039/D4NA00942H]. These structures have significantly amplified SERS signals while improving reproducibility. Mechanical deformation of the piezoelectric substrate has further boosted Raman responses. Tests at 532 nm have demonstrated over an order of magnitude enhancement across multiple analytes, indicating strong potential for diagnostic, environmental, and trace detection applications.

As Guest Editors of this themed issue, we warmly thank the authors for their excellent contributions, the anonymous reviewers for their careful evaluation and time commitment, and the *Nanoscale Advances* editorial team, especially Dr Hannah Kerr, for their consistent guidance and support in bringing this collection to completion.

